# A Novel Real-Time Reverse Transcription Loop-Mediated Isothermal Amplification Detection Platform: Application to Diagnosis of COVID-19

**DOI:** 10.3389/fbioe.2021.748746

**Published:** 2021-10-22

**Authors:** Yi Wang, Xiaoxia Wang, Hai Chen, Limei Han, Licheng Wang, Ting Chen, Sha Li, Huan Li, Yuanli Li, Zhengkun Li, Xiaoying Fu, Shaojin Chen, Mei Xing, Jun Tai, Xiong Zhu

**Affiliations:** ^1^ Experimental Research Center, Capital Institute of Pediatrics, Beijng, China; ^2^ Central and Clinical Laboratory of Sanya People’s Hospital, Sanya, China; ^3^ Wenchang People’s Hospital, Wenchang, China; ^4^ Department of Otolaryngology, Head and Neck Surgery, Children’s Hospital Capital Institute of Pediatrics, Beijing, China

**Keywords:** COVID-19, SARS-CoV-2, rRT-LAMP, reverse transcription, loop-mediated isothermal amplification

## Abstract

The ongoing Corona virus disease (COVID-19) outbreak has become a huge global health concern. Here, we reported a novel detection platform based on the loop-mediated isothermal amplification (LAMP), termed real-time reverse transcription LAMP (rRT-LAMP) and applied it for the diagnosis of COVID-19 (COVID-19 rRT-LAMP). rRT-LAMP integrates reverse transcription, LAMP amplification, restriction endonuclease cleavage and real-time fluorescence detection into one-pot reaction, and facilitates the diagnosis of COVID-19 at 64°C for only 35 min. The ORF1ab (opening reading frame 1a/b) and NP (nucleoprotein) genes of SARS-CoV-2 were detected for diagnosing COVID-19. The limit of detection (LoD) of COVID-19 rRT-LAMP assay was 14 copies (for each marker) per vessel, and no positive results were obtained from non-SARS-CoV-2 templates. To demonstrate its feasibility, a total of 33 oropharynx swab samples collected from COVID-19 patients also were diagnosed as SARS-CoV-2 infection using COVID-19 rRT-LAMP protocol. No cross-reactivity was yielded from 41 oropharynx swab samples collected from non-COVID-19 patients. These data suggesting that the COVID-19 rRT-LAMP assay is a potential detection tool for the diagnosis of SARS-CoV-2 infection in clinical, field and disease control laboratories, and will be valuable for controlling the COVID-19 epidemic.

## Introduction

The ongoing COVID-19 (Corona virus disease) epidemic caused by SARS-CoV-2 (severe acute respiratory syndrome coronavirus 2) that had previously not been documented in animals or humans, has become a major global public health concern (Huang et al., 2020). SARS-CoV-2 infection has spread rapidly to more than 200 countries/regions overseas (World Health Organization, COVID-19 Situation Report). Given the rapid spread speed (R_0_ 3.28) and mortality rate (2.3%) of SARS-CoV-2 infection, the valuable diagnostic tools are urgently required for rapidly screening suspected cases, accurately diagnosing COVID-19 and performing epidemiological surveillance.

The detection of SARS-CoV-2 RNA has been approved to be useful for the diagnosis of COVID-19, which was beneficial to preventing the spreading, controlling the sources of infection and helping patients to prevent the disease progression (Li et al., 2020; Shen et al., 2020). The spectrum of the available molecular methods for diagnosis of COVID-19 is very tight because SARS-CoV-2 is a newly emerged human coronavirus (Wu et al., 2020a). At the early stage of COVID-19 epidemic, the next-generation sequencing was employed for detecting SARS-CoV-2 RNA in various clinical specimens, while it was not available in field and clinic settings due to its longer sequencing time and high needs for equipment (Wu et al., 2020b; Zhu et al., 2021). Polymerase chain reaction (PCR)-based methodologies, including real-time PCR (RT-PCR) and real-time reverse transcription PCR (rRT-PCR) are characterized by rapid detection, high specificity and sensitivity, which have been employed for diagnosis of COVID-19 (Carter et al., 2020). However, PCR-based assays strongly rely on expensive laboratory apparatus and experienced laboratory workers, and also are time-consuming (Li et al., 2020). Unfortunately, a relatively high proportion (approximately 30%) of COVID-19 patients that were further diagnosed by chest CT were diagnosed as false negative results using the commercial COVID-19 rRT-PCR kits (To et al., 2020; Wu and McGoogan, 2020). Herein, further development of simpler, more rapid and sensitive detection tools to diagnose COVID-19 are still needed.

LAMP (Loop-mediated isothermal amplification) is the most commonly applied isothermal amplification method, and has been regarded as an attractive alternative to PCR-based methodologies due to its speed, simplicity, specificity, sensitivity and cost-effectiveness (Obande and Banga Singh, 2020). As LAMP amplification is conducted at a fixed temperature (usually between 60°C to 67°C), thus an extremely simple instrument (e.g., a water bath) is sufficient for LAMP-based assays, eliminating the use of an expensive thermal cycler. By the use of reverse transcriptase together with *Bst* 2.0 polymerase, LAMP method has been proved for amplifying and detecting RNA sequences at an isothermal step, and the reverse transcription LAMP (RT-LAMP) assay has been applied for detecting a variety of RNA viruses (Obande and Banga Singh, 2020; Shen et al., 2020). Thus, RT-LAMP assay has the potential to be a simple, rapid and reliable method for the laboratory detection of the emergence of SARS-CoV-2.

Up to now, several RT-LAMP-based methods have been developed for the diagnosis of COVID-19 (Yu et al., 2020; Zhu et al., 2020; El-Tholoth et al., 2021). However, traditional monitoring techniques (such as PH reagents, SYBR Green dyes and agarose gel electrophoresis) were employed for reporting the COVID-19 RT-LAMP results. Reporting the COVID-19 RT-LAMP results using PH reagents or SYBR Green dyes may be ambiguous when the concentration of SARS-CoV-2 RNA is lower in a clinical sample, because it is difficult to indicate weakly positive results by color change. Electrophoresis is a tedious, time-consuming procedure, and carries the huge risk of carryover contamination. Particularly, only a genetic molecule (e.g., ORF1ab) of SARS-CoV-2 was employed for establishing the COVID-19 RT-LAMP assays, and the analytical sensitivity, especially for clinical samples, may not be outstanding when compared with these LAMP-based assays using at least two genetic molecules. Herein, the novel RT-LAMP-based methods, which can overcome these shortcomings posed by the developed COVID-19 RT-LAMP assays, are in pressing demand.

Here, we reported a novel mode of RT-LAMP, termed real-time reverse transcription LAMP (rRT-LAMP), which was employed for diagnosing COVID-19 (COVID-19 rRT-LAMP). COVID-19 rRT-LAMP facilitated rapid detection of ORF1ab (opening reading frame 1a/b) and NP (nucleoprotein) genes of SARS-CoV-2 at a one-step, single-tube reaction within 35 min. This report expounds the basic rRT-LAMP principle, and validates its application for the diagnosis of COVID-19.

## Materials and Methods

### Primer Design

**FIGURE 1:**
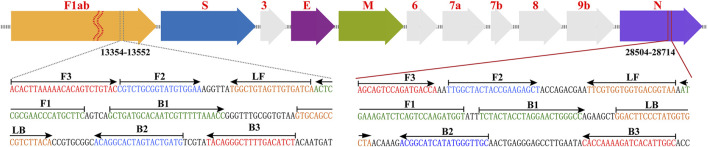
Primer design of COVID-19 rRT-LAMP method. Up row, SARS-CoV-2 genome organization (GenBank: MN908947, Wuhan-Hu-1). Bottom row, nucleotide sequence and location of ORF1ab and np gene used to design COVID-19 rRT-LAMP primers. Only part of the nucleotide sequences of ORF1ab (Left) and NP (Right) are showed. Left arrows and Right arrows show the complementary and sense sequences that are designed. The sites of primer sequence are underlined. ^*^ Note ^1^: The length of all genes is not displayed in scale (Up row). ^*^ Note ^2^: ORF1ab (Open reading frame 1a/b); S (Spike protein); E (Envelope protein); M (Membrane protein); NP (Nucleoprotein); Accessory proteins (3, 6, 7a, 7b, and 9b).

According to the rRT-LAMP principle, two LAMP primer sets (Figure 1), which targeted the ORF1ab and NP genes of SARS-CoV-2 (GenBank MN908947, Wuhan-Hu-1), were designed using PrimerExplorer V5 software (http://primerexplorer.jp/e). Each LAMP primer set, which recognizes eight different regions to amplify each gene marker, consisted of forward outer primer (F3), backward outer primer (B3), forward inner primer (FIP), back inner primer (BIP), forward loop primer (LF), and backward loop primer (LB). The specificity of the two LAMP primer sets was examined by NCBI (National Center for Biotechnology Information) BLAST, and OligoAnalyzer online software (Version 3.1, Integrated DNA Technologies, Coralville, IA) was employed for secondary structure and primer dimer analysis. Details of ORF1ab- and NP-LAMP primer sets, including sequences, locations and modifications, were listed in Supplementary Table S1; Figure 1. All of the oligomers were synthesized by Tianyi-Huiyuan Biotech. Co., Ltd. (Beijing, China), and purified at HPLC grade.

### rRT-LAMP Reaction

The rRT-LAMP (ORF1ab- and NP-rRT-LAMP) was carried out in a one-step 25 μL reaction mixture containing 12.5 μL 2 × isothermal reaction buffer (Huidexin Biotechnology. Co., Ltd. Tianjin, China) [40 mM Tris-HCl (pH 8.8), 40 mM KCl, 16 mM MgSO_4_, 20 mM (NH_4_)_2_SO_4_, 2 M betaine and 0.2 % Tween-20], 8 U of Bst 2.0 DNA polymerase, 5 U of avian myeloblastosis virus reverse transcriptase V3.0 (AMV, Takara), 1 U Nb. BsrDI (New England Biolabs), 1.4 mM dATP, 1.4 mM dCTP, 1.4 mM dGTP, 1.4 mM dTTP, 0.4 μM each of F3 and B3, 0.8 μM each of LF* and LB, 1.6 μM each of FIP and BIP, and template (1 μL for the standard plasmid/5 µL for clinical samples). Real-time fluorescence, real-time turbidity (LA-320C) and agarose gel electrophoresis were employed for reporting the rRT-LAMP reactions and for optimizing the amplification parameters (e.g. assay’s temperature and time).

### Sensitivity of the rRT-LAMP Assay

Two standard plasmids (named as ORF1ab-plasmid and NP-plasmid) were commercially constructed by Tianyi-Huiyuan Biotech. Co., Ltd. (Beijing, China), which contain the ORF1ab and NP sequences, respectively. The initial concentrations of ORF1ab- and NP-plasmids were 1.4 × 10^8^ copies/ µl, then ten-fold serial dilutions (1.4×10^5^ to 1.4 × 10^−1^ copies) of ORF1ab-plasmid and NP-plasmid were prepared. The serial dilutions of ORF1ab-plasmid and NP-plasmid were used for defining the limit of detection (LoD) of COVID-19 rRT-LAMP, and a volume of 1 µL of these templates were used for COVID-19 rRT-LAMP reactions.

### Specificity of the COVID-19 rRT-LAMP Assay

The specificity of the COVID-19 rRT-LAMP assay was evaluated using various templates, including synthetic nucleic acid sequences, viruses, bacteria and fungi (Supplementary Table S2).

### Feasibility of COVID-19 rRT-LAMP Using Clinical Samples

A total of 33 respiratory samples (pharyngeal/nasal swabs), which were collected from 33 COVID-19 patients (Sanya People’s Hospital, Hainan) (Table 1), were defined according to standard diagnostic and treatment criteria of COVID-19 (Trial Version 6). The pharyngeal and nasal swab samples were collected using a Flocked sterile plastic swab applicator, which was placed in a Universal Viral Transport Medium (UVIM) for viruses (HiDNA biotech. Co., Ltd.). Particularly, aliquots (200 µL) of UVIM (pharyngeal and nasal swab samples) were subjected to extract the RNA templates, and the process only required 15 min using a rapid RNA Extraction Kit (Daan Nucleic Acid Isolation Kit, Daanene Co. LtD.) with an automatic instrument (Smart 32, Daanene Co. LtD.). These templates were firstly used for clinical and laboratory diagnosis, which was performed using two RT-qPCR kits (Daangene Co. LtD. and BGI Co. LtD.) as recommended by the China CDC and Hainan CDC. Then, a volume of 5 μL of templates was used as the input template for COVID-19 rRT-LAMP test.

**TABLE 1:** Detection results of COVID-19 rRT-LAMP for pharyngeal swab samples.

Sample number	Gender	Age	Judgment result of RT-PCR I	Result of RT-PCR I		Judgment result of RT-PCR II	Result of RT-PCR II		Judgment result of rRT-LAMP	Result of rRT-LAMP		Clinical diagnosis^a^
				ORF1ab	NP		ORF1ab	NP		ORF1ab	NP	
S1	F	36	Positive	+	+	Positive	+	+	Positive	+	+	+
S2	F	56	Positive	+	+	Positive	+	+	Positive	+	+	+
S3	M	26	Positive	+	+	Positive	+	+	Positive	+	+	+
S4	M	25	Positive	+	+	Positive	+	+	Positive	+	+	+
S5	M	62	Positive	+	—	Positive	+	—	Positive	+	—	+
S6	F	65	Positive	—	+	Positive	—	+	Positive	—	+	+
S7	F	65	Positive	+	+	Positive	+	+	Positive	+	+	+
S8	M	73	Positive	+	+	Positive	+	+	Positive	+	+	+
S9	M	71	Positive	+	+	Positive	+	+	Positive	+	+	+
S10	F	31	Positive	+	+	Positive	+	+	Positive	+	+	+
S11	M	71	Positive	+	+	Positive	+	+	Positive	+	+	+
S12	M	62	Positive	+	+	Positive	+	+	Positive	+	+	+
S13	F	76	Positive	+	+	Positive	+	+	Positive	+	+	+
S14	M	39	Positive	+	+	Positive	+	+	Positive	+	+	+
S15	M	41	Positive	+	—	Positive	+	—	Positive	+	—	+
S16	F	65	Positive	+	+	Positive	+	+	Positive	+	+	+
S17	M	62	Positive	+	+	Positive	+	+	Positive	+	+	+
S18	F	38	Positive	+	+	Positive	+	+	Positive	+	+	+
S19	M	39	Positive	+	+	Positive	+	+	Positive	+	+	+
S20	M	73	Positive	+	+	Positive	+	+	Positive	+	—	+
S21	F	56	Positive	+	+	Positive	+	+	Positive	+	+	+
S22	M	71	Positive	+	+	Positive	+	+	Positive	+	+	+
S23	F	38	Negative	—	+	Positive	—	+	Positive	—	+	+
S24	F	46	Negative	—	+	Positive	+	+	Positive	—	+	+
S25	F	37	Positive	+	+	Positive	+	+	Positive	+	+	+
S26	M	73	Positive	+	+	Positive	+	+	Positive	+	+	+
S27	M	31	Negative	—	—	Positive	—	+	Positive	—	+	+
S28	F	42	Positive	+	+	Positive	+	+	Positive	+	+	+
S29	M	65	Positive	—	+	Positive	—	+	Positive	+	+	+
S30	F	36	Positive	+	+	Positive	+	+	Positive	+	+	+
S31	M	68	Positive	—	—	Positive	—	+	Positive	—	+	+
S32	M	37	Positive	—	+	Positive	—	+	Positive	—	+	+
S33	F	56	Positive	+	—	Positive	+	+	Positive	+	—	+

a The “+” represent the patient of COVID-19.

Collection and analysis of these RNA templates were approved by Sanya People’s Hospital (Ethical approval: SYPH-2019[41]-2020-03-06).

## Results

### Rational Design of rRT-LAMP Design

**FIGURE 2:**
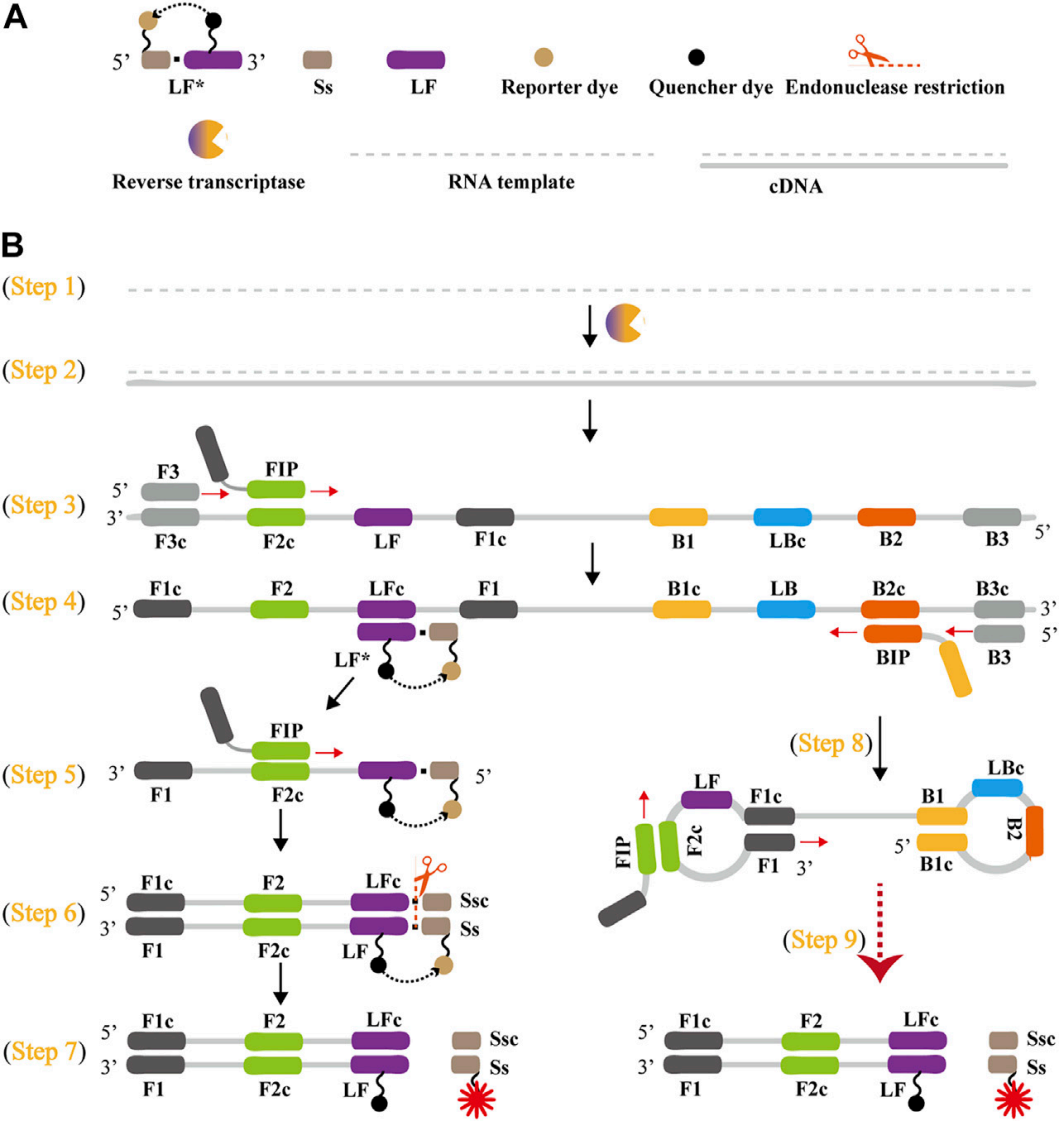
Outline of rRT-LAMP assay. **(A)** Schematic depiction of a new forward loop primer (LF*)/backward loop primer (LB*). LF*/LB* is an extension of the conventional LF/LB with an endonuclease recognition site (Ss, its complementary sequence named Ssc) at the 5′ end. LF*/LB* is modified with a reporter at the 5′-end, and is labeled with quencher dye in the middle. **(B)**, Outline of rRT-LAMP technology. Six primers, including forward displacement primer (F3), backward displacement primer (B3), forward inner primer (FIP), backward inner primer (BIP), LB and new LF*, are displayed for illustrating the rRT-LAMP principle. .

The schematic reaction mechanism of rRT-LAMP is exhibited in Figure 2. The rRT-LAMP system integrates reverse transcription, isothermal amplification, restriction endonuclease digestion and real-time fluorescence detection into a one-pot reaction mixture. In particular, a loop primer (LF or LB) is modified with short sequence (named as Ss) at the 5′ end, and the new LF or LB is named as LF* or LB*. The Ss can be recognized by NB. *bsrDI* enzyme. Then, LF* or LB* is labeled at 5′ end with a reporter dye and in the middle with a corresponding dark quencher (Figure 2A). The reporter molecule and quenching dye are very close to each other, thus this successfully prevents the emitted fluorescence of the reporter dye.

A Nb. *BsrDI* enzyme is employed for achieving the rRT-LAMP reaction, because it is able to specially recognize the short sequence (Ss) 5′-GCAATGNN-3' (N = A, G, C and T) and cleavages Ss 5′-GCAATG-3′ at a constant temperature (60°C-65 °C). Thus, Ss (5′-GCAATG-3′) is added to the 5′ end to construct LF* or LB*, and an additional base (T) also is added to the 5′ end of Ss to protect the recognition site. As a result, the LF* or LB* primer maintains its function as a loop primer with the added advantage of simultaneous detection of the rRT-LAMP reactions by release of quenching (Figure 2B).

The outline of rRT-LAMP assay with LF* or LB* is depicted in Figure 2B. For clarity, the LB* primer is not displayed. In the rRT-LAMP system, a total of six primers, including F3, B3, FIP, BIP, LF* and LB, was used. The RNA templates were firstly converted to cDNA with the assistance of reverse transcriptase (e.g., avian myeloblastosis virus reverse transcriptase, AMV), and then the cDNA serves as the initial template for subsequent LAMP reaction (Figure 2B **,** step 1 and 2). During the LAMP amplification stage, FIP hybridized to F2c in the target sequence and initiated complementary strand synthesis (Figure 2B, step 3). The primer F3 then hybridizes to F3c in the target sequence, which initiates strand displacement synthesis (Figure 2B, step 3). Thus, the newly synthesized strand derived from FIP primer is displaced by the F3 primer synthesis, producing a single strand (Figure 2B, Step 3). This single-stranded DNA serves as templates for subsequent LF*-primed strand displacement DNA synthesis, BIP-initiated DNA synthesis and B3-primed strand displacement synthesis (Figure 2B, Step 4). As a result, the strand displacement polymerase (*Bst 2.0*) extends in tandem generating two different products. In particular, the LF* strand acts as the template for subsequent extension by FIP (Figure 2B, Step 5), and the new double-stranded terminal sequence (Ss and its complementary Ssc sequences) are cleaved by NB. *bsrDI* enzyme (Figure 2B, Step 6), resulting a gain of fluorescence signal (Figure 2B, Step 7). Furthermore, the BIP strand (from Step 4) forms a dumbbell-shaped product, which is rapidly converted to a stem-loop form by self-primed synthesis. This stem-loop form then serves as the starting templates for subsequent amplification cycling, the second stage of the LAMP amplification (elongation and cycling steps) (Figure 2, step 8). The subsequent exponential amplification also releases the quenching, which gives rise to additional release of fluorophores, resulting in exponential signal detection.

### Rational Design of COVID-19 rRT-LAMP Design

**FIGURE 3:**
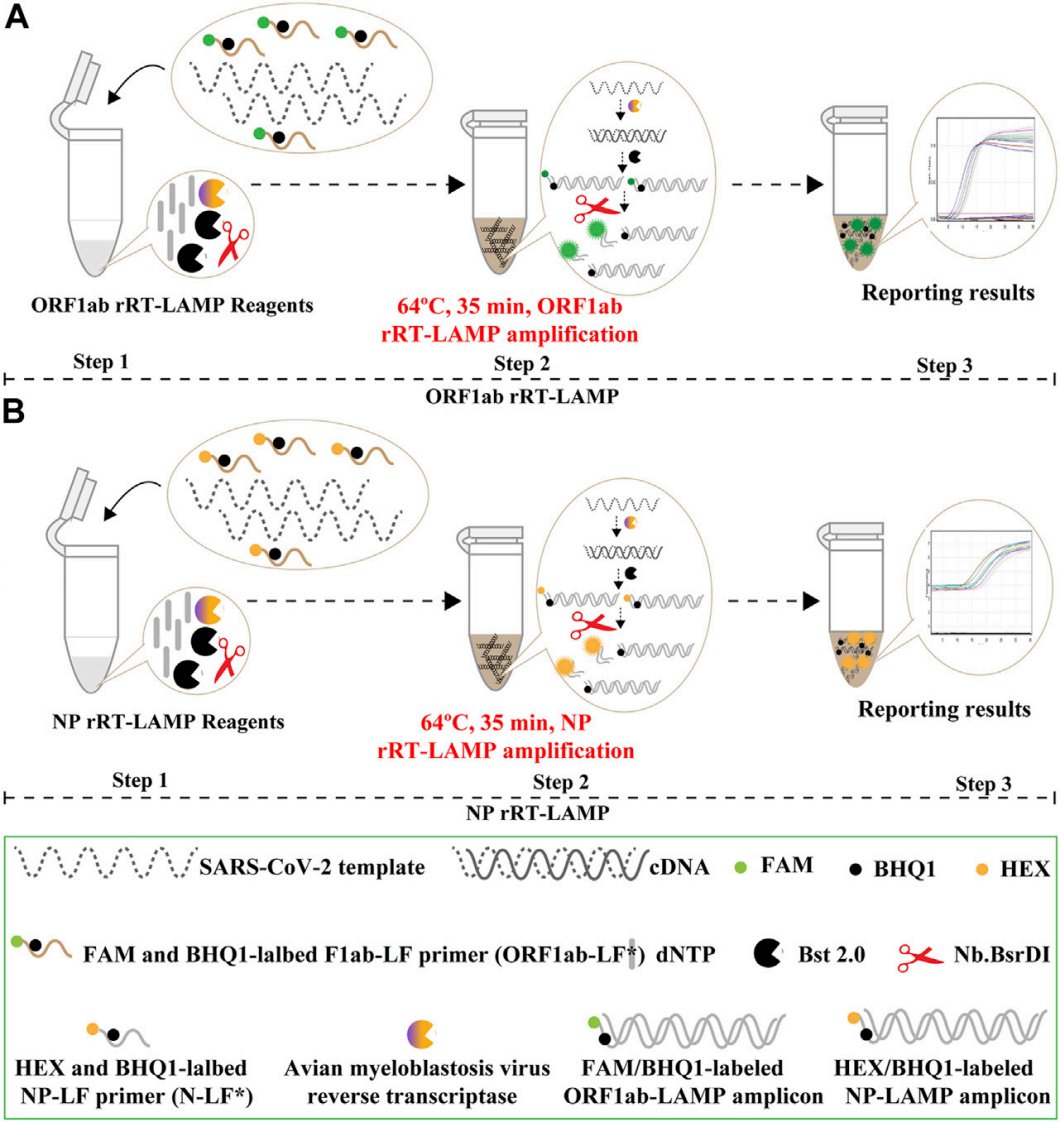
Mechanistic description of the COVID-19 rRT-LAMP assay. **(A)**. rRT-LAMP for ORF1ab detection. **(B)**. rRT-LAMP assay for NP detection. Step 1, Preparing the COVID-19 rRT-LAMP amplification mixtures. Step 2, COVID-19 rRT-LAMP reaction. Step 3, The result interpretation of the COVID-19 rRT-LAMP reaction.

The COVID-19 rRT-LAMP design scheme is depicted in Figure 3. In the COVID-19 rRT-LAMP system, one reported dye, FAM (6-carboxy-fluorescein) is modified at the 5′ end of ORF1ab-LF* primer (Figure 3A), another, HEX (Hexachloro-fluorescein) is labeled to the 5′ end of NP-LF* primer (Figure 3B). To emit fluorescence of the reporter molecule, the quenching dye (Black Hole Quencher-1, BHQ1) is modified in the middle of the ORF1ab-LF* and NP-LF* primers. Thus, the ORF1ab-LF* is labeled simultaneously with FAM and BHQ1, NP-LF* for HEX and BHQ1 (Figure 3, Step 1). The SARS-CoV-2 RNA firstly is converted to cDNA with the assistance of reverse transcriptase AMV, and then serves as the initial template for subsequent LAMP amplification (Figure 3, Step 2). During the LAMP amplification stage, the terminals of double-stranded products derived from ORF1ab-LF* and NP-LF* primers can be digested by the NB. *bsrDI* enzyme, thus the reporter dyes (FAM and HEX) are separated from the quenching dye (BHQ1) leading to the gain of fluorescent signals (Green and Yellow signals) (Figure 3, Step 3). The fluorescent signals released from COVID-19 rRT-LAMP reactions can be detected by a real-time system (e.g., Genie III).

### Validation of ORF1ab- and NP-RT-LAMP Amplifications

To confirm the correct amplification of rRT-LAMP assay, we performed the ORF1ab- and NP-rRT-LAMP reactions in the presence or absence of plasmid templates. Using real-time detection, the release of quenching was obtained as a robust increase of FAM (Green channel) and HEX (Yellow channel) signals in the positive results, but not in negative and blank controls **(** Supplementary Figures S1A,C, Left row). Then, the rRT-LAMP products were electrophoresed to verify the presence of the expected ladder bands (Supplementary Figures S1B,D, Right row). Hence, each set of rRT-LAMP primers designed in this study could amplify the predicted product specifically from the templates of the corresponding plasmids. These results suggested that the rRT-LAMP method using ORF1ab- and NP-LAMP primer sets could be applied for the detection of SARS-CoV-2. Furthermore, the optimal reaction temperature for COVID-19 rRT-LAMP was also confirmed with 64 °C shown to be the best for COVID-19 rRT-LAMP reaction (Supplementary Figures S2, S3).

### Analytical Sensitivity of COVID-19 rRT-LAMP Assay

**FIGURE 4:**
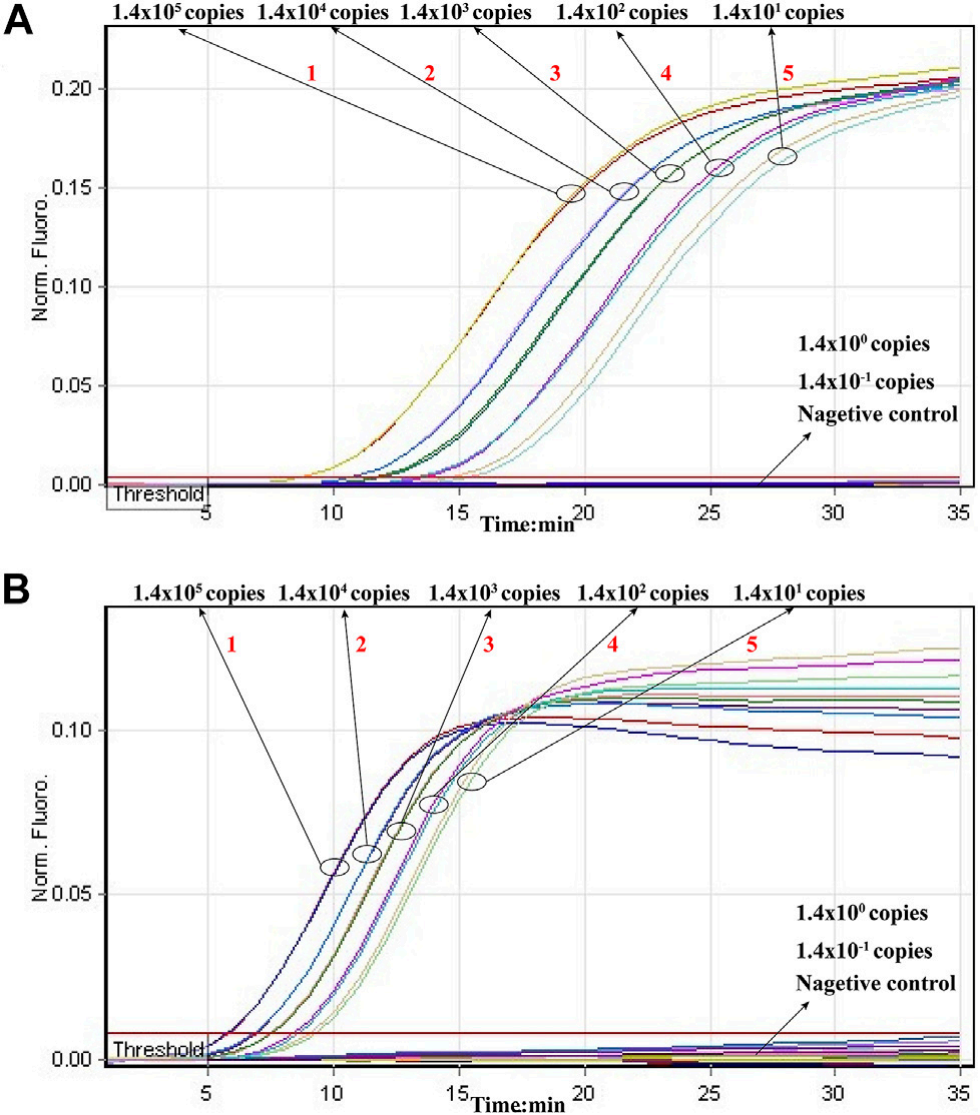
Sensitivity of rRT-LAMP assay. Two sets of rRT-LAMP primer targeting ORF1ab and NP genes were in different reactions: ORF1ab [**(A)**, FAM channel] and NP [**(B)**, Hex channel]. The template levels from 1.4 × 10^5^ copies to 1.4 × 10^1^ copies per tube generated the positive signals, 1.4 × 10^0^ copies and 1.4 × 10^−1^ copies per reaction and negative control generated negative signal.

Then, we tested the rRT-LAMP’s sensitivity. rRT-LAMP amplified ORF1ab and NP genes with pure templates from ORF1ab-plasmid and NP-plasmid, respectively. The fluorescent intensity-reaction time curves synchronized well among two replicates containing same dilution of target plasmids. The release of quenching was generated from 1.4 × 10^5^ copies to 1.4 × 10^1^ copies of plasmid templates, and the FAM and HEX signals corresponded to ORF1ab and NP detection, respectively (Figures 4A,B). Thus, the analytical sensitivity of rRT-LAMP for ORF1ab and NP detection was 1.4 × 10^1^ copies per reaction.

**FIGURE 5:**
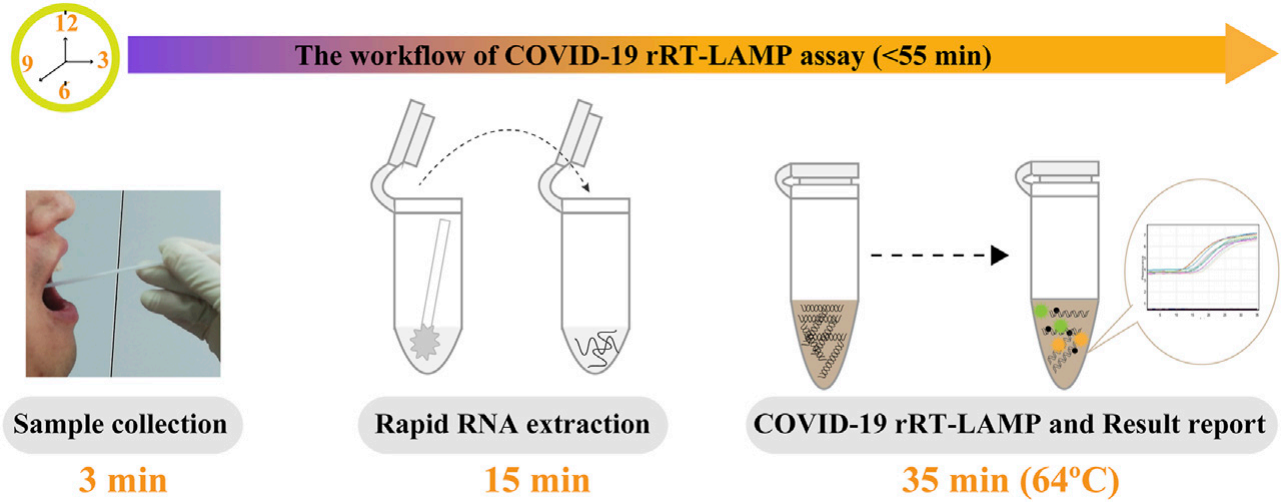
The workflow of COVID-19 rRT-LAMP assay. Three steps, including sample collection (3 min), rapid RNA preparation (15 min), rRT-LAMP reaction and result reporting (35 min), were need for performing the COVID-19 rRT-LAMP diagnosis test, and the whole procedure could be finished within 55 min.

Particularly, the positive results can be observer in as short as 10 min (Figure 4), and only 35 min are required for COVID-19 rRT-LAMP test. Thus, the whole diagnostic test, including sample collection (3 min), rapid SARS-CoV-2 RNA extraction (15 min), rRT-LAMP reaction and results reporting (35 min), can be finished within 55 min (Figure 5).

### Specificity of the COVID-19 rRT-LAMP Technology

**FIGURE 6:**
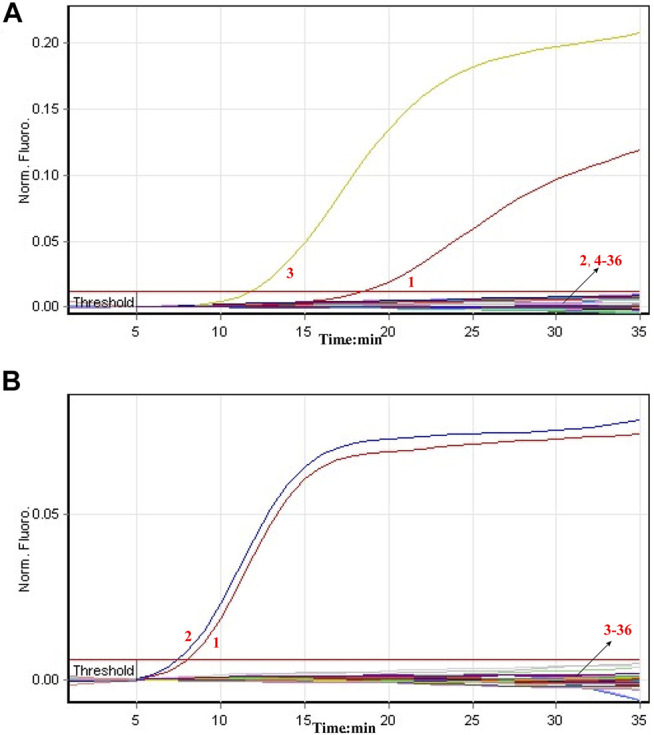
Specificity of COVID-19 rRT-LAMP assay. The COVID-19 rRT-LAMP reactions were carried out using different genomic templates and the results were monitored by means of real-time analysis. **(A)** and **(B)** were produced from FAM (Green) and HEX (Yellow) channels, respectively. Signal 1, positive control (1.4 × 10^2^ copies of ORF1ab- and NP-plasmids); Signal 2, positive control (1.4 × 10^2^ copies of NP-plasmids); Signal 3, positive control (1.4 × 10^2^ copies of ORF1ab-plasmids); Signals 4-36, coronavirus HKU1 (ORF1ab), coronavirus HKU1 (NP), coronavirus 229E (ORF1ab), coronavirus 229E (NP), coronavirus NL63 (ORF1ab), coronavirus NL63 (NP), coronavirus OC43 (ORF1ab), coronavirus OC43 (NP), coronavirus SARS (ORF1ab), coronavirus SARS (NP), coronavirus MERS (ORF1ab), coronavirus MERS (NP), *Infuenza virus A* (H1N1), *Infuenza virus A* (unidentified subtype), *Infuenza virus B* (unidentified subtype), *Parainfluenza Virus*, *Human Adenovirus*, *Syncytial Virus*, *Human enterovirus* (EV71), Coxsackievirus (CAV16), *Mycoplasma*, *Chlamydia*, *Lpneumophila*, *Pseudomonas aeruginosa*, *Klebsiella pneumoniae*, *Neisseria meningitidis*, *Acinetobacter baumannii*, *Staphylococcus aureus*, *Staphylococcus saprophytics*, *Candida tropicalis*, *Cryptoccus neo formas*, *Streptococcus pneumona*, *Candida albicans*.

The specificity of COVID-19 rRT-LAMP assay was tested in relation to data with nucleic acids from synthetic nucleic acid sequences, viruses, bacteria and fungi templates (Supplementary Table S2). The positive fluorescence signals were obtained only when nucleic acids of ORF1ab-plasmid and NP-plasmid were applied as the templates for COVID-19 rRT-LAMP analysis, and the target sequences could be by correctly identified (Figure 6). As expected, no positive signals were observed with any of the nucleic acids from non-ORF1ab-plasmid and non-NP-plasmid templates. These results indicated that the specificity of COVID-19 rRT-LAMP methodology was 100% in this study.

### Evaluation of the COVID-19 rRT-LAMP Methodology in

A total of 74 clinical samples, which were initially diagnosed using rRT-RCR assays, were collected in Sanya People’s Hospital in 2020. Particularly, 33 were COVID-19 positive samples, and 41 samples were diagnosed with pneumonia and confirmed to be caused by non-SARS-CoV-2 pathogens (e.g., *Mycoplasma pneumoniae*, *Pseudomonas aerugiosa*, *Klebsiella pneumoniae*, infuenza virus A and B etc.). The templates were leftover RNA extracted from 74 clinical samples after rRT-PCR analysis. Using COVID-19 rRT-LAMP assay, 33 COVID-19 samples also were diagnosed as SARS-CoV-2 infection (Table 1), and no positive results were obtained from 41 COVID-19 negative samples. These data preliminarily demonstrated that the COVID-19 rRT-LAMP methodology had a highly analytical sensitivity and specificity for the diagnosis of COVID-19.

## Discussion

The ongoing outbreak of COVID-19 poses a huge global public health concern (Heymann and Shindo, 2020; Hui et al., 2020). Due to the lack of effective antiviral drugs or vaccines for COVID-19, rapid, reliable and early diagnostic technologies for SARS-CoV-2 is of top priority for achieving public health interventions that can decrease or prevent further spread of COVID-19 (Wu et al., 2020c). Importantly, such diagnostic tests are needed not only in regions/countries where COVD-19 is already spreading but also in regions/countries where COVID-19 has not yet occurred.

To achieve more such effective diagnostic technologies, we successfully devised a novel LAMP-based assay that offers simple, rapid and reliable diagnosis for SARS-CoV-2 infection. COVID-19 rRT-LAMP simultaneously integrates reverse transcription, LAMP amplification, restriction endonuclease cleavage and real-time fluorescence detection into one-pot reaction, and achieves the detection of SARS-CoV-2 RNA at a constant temperature. Our diagnostic test only requires a relatively simple fluorescent instrument (e.g., Genie III) to maintain a fixed temperature (64°C) for 35 min. Comparing with these COVID-19 RT-LAMP techniques developed by previous reports, the rRT-LAMP results were detected by real-time fluorescence analysis, which eliminates the requirements of special reagents (e.g., pH indicators), complex processes (e.g., electrophoresis) and expensive instruments (e.g., real-time turbidity) (Lamb et al., 2020; Yu et al., 2020). The whole detection procedure can be finished within 55 min including sample collection (3 min), rapid SARS-CoV-2 RNA preparation (15 min), rRT-LAMP amplification and result interpretation (35 min). Regarding these traits, the COVID-19 rRT-LAMP methodology is a technically simple, rapid and economical technique, which provides practical solutions for clinical, field and disease control laboratories, especially in low-resource settings.

Two sets of rRT-LAMP primer, including ORF1ab-rRT-LAMP and NP-rRT-LAMP primer sets, were specifically designed according to the rRT-LAMP principle. The primer sets of ORF1ab- and NP-rRT-LAMP contain six primers (ORF1ab/NP-F3, ORF1ab/NP-B3, ORF1ab/NP-FIP and ORF1ab/NP-BIP), which recognize eight regions within the ORF1ab and NP genes, respectively (Figure 1). Thus, rRT-LAMP primer sets ensure that our COVID-19 rRT-LAMP has high selectivity for target sequence detection. The data produced from specificity tests suggested that all positive results were obtained from positive control and SARS-CoV-2 RNA templates (Figure 6; Table 1), and all negative results were yielded from non-SARS-CoV-2 templates. Hence, our technology could effectively prevent any false-positive or false-negative results from current SARS-CoV-2 RT-LAMP methods that only amplify a single genetic target (e.g., ORF1ab gene) (Lamb et al., 2020; Yu et al., 2020).

The analytical sensitivity of COVID-19 rRT-LAMP methodology also is sufficient for the detection of SARS-CoV-2. Our data revealed that COVID-19 rRT-LAMP assay was able to detect down to 14 copies each of the targets (ORF1ab- and np-plasmids) (Figure 4). Particularly, we did not compare the sensitivity result yielded from commercial rRT-PCR kits with COVID-19 rRT-LAMP assay, because the quality of commercial COVID-19 rRT-PCR kits used in Sayan People’s Hospital remains uneven. These rRT-PCR kits generated inconsistent sensitivity results when they were used for detecting the 10-fold serially diluted ORF1ab-plasmid NP-plasmid templates.

For the analysis of clinical samples, the COVID-19 rRT-LAMP assay showed high sensitivity and specificity, and able to correctly diagnose 100% (33/33) of COVID-19 samples determined by rRT-PCR (Table 1) and 100% (41/41) of samples from non-COVID-19 patients. The concordance of high reliability between our assay and commercial rRT-PCR kits for the diagnosis of COVID-19 was preliminarily demonstrated using 74 clinical samples. Some factors may contribute to this. The COVID-19 rRT-LAMP technology, as a novel LAMP-based methodology, was less affected by the presence of various inhibitors and salts, or able to tolerate the inhibitory effect of various nucleic acids in the reaction mixtures (Kaneko et al., 2007). Simultaneously, some factors may affect COVID-19 rRT-LAMP performance. The genomic templates of SARS-CoV-2 are the RNA, which is extremely sensitive to degradation by postmortem procedures, inadequate sample handing or storage. Hence, the quality of target templates (such as integrity and purity) is a key factor for the success of COVID-19 rRT-LAMP diagnosis. In addition, other elements may affect COVID-19 rRT-LAMP diagnostic results including sampling timing (different periods of the disease development) or specimen’s source (lower or upper respiratory tract).

## Conclusion

A novel diagnostic test, termed rRT-LAMP, was successfully devised and applied to diagnosis of COVID-19 (COVID-19 rRT-LAMP). COVID-19 rRT-LAMP was preliminarily validated using pure nucleic acid templates and clinical samples. The whole test procedure from sample collection to result interpretation could be completed within 55 min. The relatively simple instrument was required, which makes it feasible to conduct COVID-19 diagnosis in various laboratories. The rapidity, feasibility, sensitivity, high specificity, its low cost and ease of use make the COVID-19 rRT-LAMP assay a promising test tool for application in public health, disease control and clinic laboratories.

## Data Availability

The original contributions presented in the study are included in the article/Supplementary Material, further inquiries can be directed to the corresponding authors.
