# Supplementary material for: A Novel Real-Time Reverse Transcription Loop-Mediated Isothermal Amplification Detection Platform: Application to Diagnosis of COVID-19
Source: Front Bioeng Biotechnol. 2021 Oct 22;9:748746. doi: 10.3389/fbioe.2021.748746 (PMC8569142; doi:10.3389/fbioe.2021.748746)
Supplement: Supplementary file 1 [file DataSheet1.docx]

**Supplementary Materials**:

**A novel real-time reverse transcription loop-mediated isothermal amplification detection platform: Application to diagnosis of COVID-19**

Yi Wang ^1,*, †^, Xiaoxia Wang ^2,*^, Hai Chen ^2^, Limei Han ^2^, Licheng Wang ^2^, Ting Chen ^2^, Sha Li ^2^, Huan Li ^2^, Yuanli Li ^2^, Zhengkun Li ^2^, Xiaoying Fu ^2^, Shaojin Chen ^2^, Mei Xing ^3^, Jun Tai ^4, †^ and Xiong Zhu ^4, †^

^1^ Experimental Research Center, Capital Institute of Pediatrics, Beijng 100020, P. R. China.

^2^ Central & Clinical Laboratory of Sanya People’s Hospital, Sanya, Hainan 572000, People’s Republic of China.

^3^ Wenchang People's Hospital, Wenchang, Hainan 572000, P. R. China.

^4^ Department of Otolaryngology, Head and Neck Surgery, Children's Hospital Capital Institute of Pediatrics, Beijing 100020, P. R. China.

**Short title**: COVID-19 rRT-LAMP assay

**Figure**: 6

**Tables**: 1

**Supplementary Materials**: 1

† Correspondence: **Yi Wang (**E-mail: [wildwolf0101@163.com](mailto:wildwolf0101@163.com) **)**

Experimental Research Center, Capital Institute of Pediatrics, Beijng 100020, P. R. Chin

† **Jun Tai (**trenttj@163.com**)**

Department of Otolaryngology, Head and Neck Surgery, Children's Hospital Capital Institute of Pediatrics, Beijing 100020, P. R. China.

† **Xiong Zhu (**trenttj@163.com**)**

- *These authors contributed equally to this article.
- **Tables (Supplementary materials)**
- **Table S1.** The primers used in this study

| - **Primers name ^a^** | - **Sequences and modifications ^b^** | - **Length ^c^** |
| --- | --- | --- |
| - F1ab-F3 | - 5'-ACACTTAAAAACACAGTCTGTAC-3' | - 23 nt |
| - F1ab-B3 | - 5'-AGATGTCAAAAGCCCTGTA-3' | - 19 nt |
| - F1ab-FIP | - 5'-GAAGCATGGGTTCGCGGAGTCGTCTGCGGTATGTGGAA-3' | - 38 mer |
| - F1ab-BIP | - 5'-GCTGATGCACAATCGTTTTTAAACCCATCAGTACTAGTGCCTGT-3' | - 44 mer |
| - F1ab-LF* | - 5'-FAM-TGCAATG-TGAT(BHQ1)CACAACTACAGCCAT-3' | - 26 nt |
| - F1ab-LB | - 5'-GTGCAGCCCGTCTTACA-3' | - 17 nt |
| - NP-F3 | - 5'-AGCAGTCCAGATGACCA-3' | - 17 nt |
| - NP-B3 | - 5'-GCCAATGTGATCTTTTGGTG-3' | - 20 nt |
| - NP-FIP | - 5'-ACCATCTTGGACTGAGATCTTTCATTTGGCTACTACCGAAGAGCT-3' | - 45 mer |
| - NP-BIP | - 5'-TCTACTACCTAGGAACTGGGCCGCAACCCATATGATGCCGT-3' | - 41 mer |
| - NP-LF* | - 5'-Hex-TGCAATG-TT(BHQ1)ACCGTCACCACCACGAA-3' | - 26 nt |
| - NP-LB | - 5'-GGACTTCCCTATGGTGCTA-3' | - 19 nt |

- ^a^ F1ab, open reading frame 1a/b; N, nucleoprotein gene;
- ^b^ FAM, 6-carboxy-fluorescein; HEX, Hexachloro fluorescein; BHQ1, black hole quencher 1.
- ^c^ mer, monomeric unit; nt, nucleitide.
- **Table S2. The pathogen used in this study**

| - **Strains** | - **Serotypes (subtypes) ^a^** | - **No. of strains** | - **RT-LAMP-NBS ^b^** |
| --- | --- | --- | --- |
| - Positive control | - Unidentified | - 1 | - P |
| - *Coronavirus* | - HKU1 (F1ab) | - 1 | - N |
| - *Coronavirus* | - HKU1 (NP) | - 1 | - N |
| - *Coronavirus* | - 229E (F1ab) | - 1 | - N |
| - *Coronavirus* | - 229E (NP) | - 1 | - N |
| - *Coronavirus* | - OC43 (F1ab) | - 1 | - N |
| - *Coronavirus* | - OC43 (NP) | - 1 | - N |
| - *Coronavirus* | - NL63 (F1ab) | - 1 | - N |
| - *Coronavirus* | - NL63 (NP) | - 1 | - N |
| - *Coronavirus* | - SARS (F1ab) | - 1 | - N |
| - *Coronavirus* | - SARS (NP) | - 1 | - N |
| - *Coronavirus* | - MERS (F1ab) | - 1 | - N |
| - *Coronavirus* | - MERS (NP) | - 1 | - N |
| - *Infuenza Virus A* | - H1N1 | - 1 | - N |
| - *Infuenza Virus A* | - Unidentified | - 8 | - N |
| - *Infuenza Virus B* | - Unidentified | - 3 | - N |
| - *Parainfluenza Virus* | - Unidentified | - 1 | - N |
| - *Human Adenovirus* | - Unidentified | - 2 | - N |
| - *Syncytial Virus* | - Unidentified | - 1 | - N |
| - *Human enterovirus* | - EV71 | - 1 | - N |
| - Coxsackievirus | - CAV16 | - 1 | - N |
| - *Mycoplasma* | - Unidentified | - 31 | - N |
| - *Chlamydia* | - Unidentified | - 2 | - N |
| - *Lpneumophila* | - Unidentified | - 8 | - N |
| - *Pseudomonas aeruginosa* | - Unidentified | - 1 | - N |
| - *Klebsiella pneumoniae* | - Unidentified | - 1 | - N |
| - *Neisseria meningitidis* | - Unidentified | - 1 | - N |
| - *Acinetobacter baumannii* | - Unidentified | - 1 | - N |
| - *Staphylococcus aureus* | - Unidentified | - 1 | - N |
| - *Staphylococcus saprophytics* | - Unidentified | - 1 | - N |
| - *Candida tropicalis* | - Unidentified | - 1 | - N |
| - *Cryptoccus neo formas* | - Unidentified | - 1 | - N |
| - *Streptococcus pneumona* | - Unidentified | - 1 | - N |
| - *Candida albicans* | - Unidentified | - 1 | - N |

- a F1ab, opening reading frame 1a/b; NP, nucleoprotein. These coronavirus nucleic acid sequences, including HKU1 (F1ab), HKU1 (NP), 229E (F1ab), 229E (NP), NL63 (F1ab), NL63 (NP), OC43 (F1ab), OC43 (NP), SARS (F1ab), SARS (NP), MERS (F1ab) and MERS (NP), were synthesized by Tianyi-Huiyuan Biotech (Beijing, China).
- ^b^ Only positive control (1.4x10^2^ copies each of F1ab-plasmid and NP-plsmid) could be detected by the COVID-19 rRT-LAMP methodology.

**Figure legends (Supplementary materials)**

- **
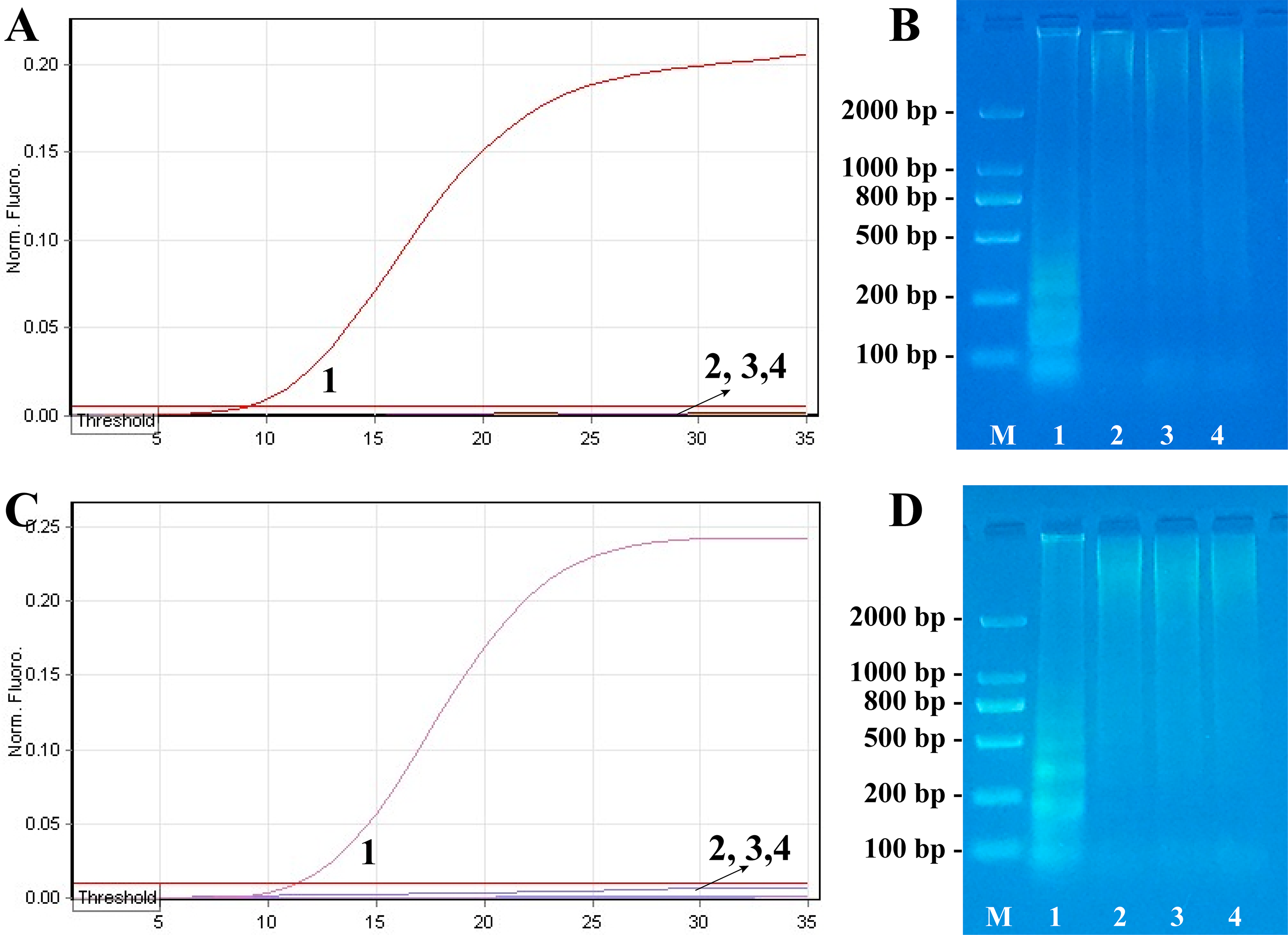
**
- **Figure S1. Confirmation of F1ab- and NP-rRT-LAMP reactions**
- **A** and **C**, the rRT-LAMP was analyzed by means of real-time format, and the two figures were obtained from FAM (labeling LF* of F1ab sequence) and HEX (labeling LF* of NP gene) channels. Signals A1 and C1 indicate F1ab-plasmid and NP-plasmid templates in FAM and HEX channels, respectively, and signals A2/C2, A3/C3 and A4/C4 indicate negative control (H1N1), negative control (CAV16) and blank control (DW). **B** and **D**, agarose gel electrophoresis applied to rRT-LAMP products; lane 1, DL 100-bp DNA markers, lane B2 and D2, positive F1ab-rRT-LALP and NP-rRT-LAMP products, respectively; lane B3/D3, lane B4/D4 and lane B5/D5, negative control (H1N1), negative control (CAV16) and blank control (DW).
- Note*: H1N1, Influenza H1N1 virus; CVA16, Coxsackievirus A16; DW, Distilled water.
-
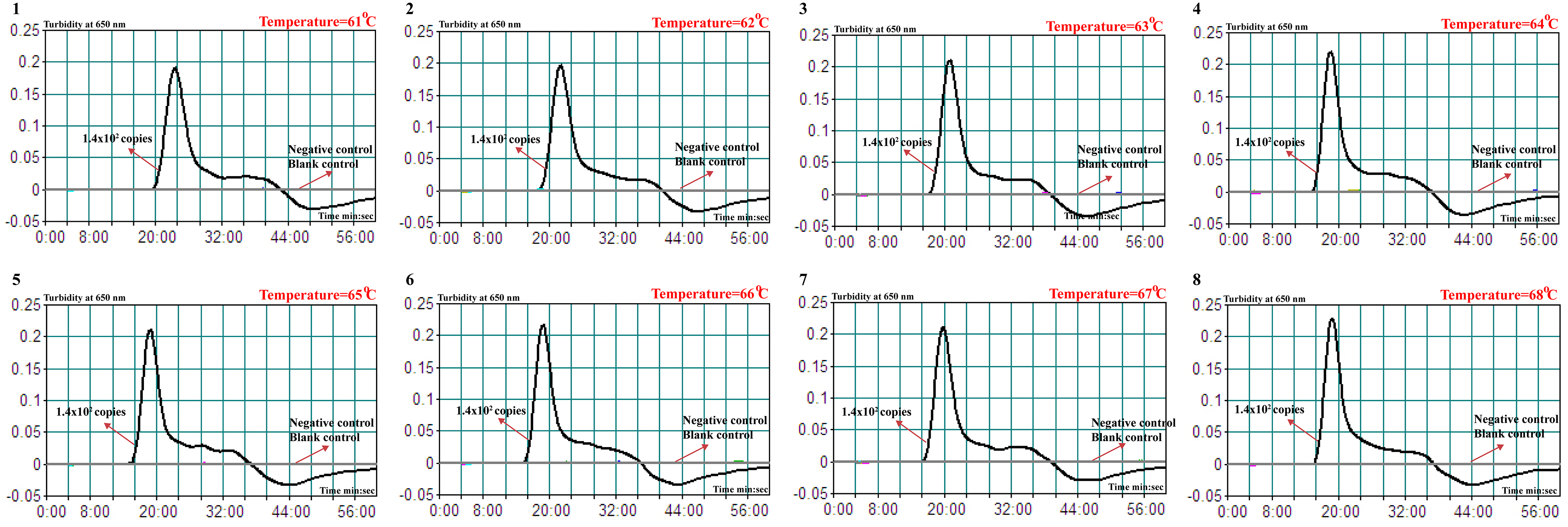

- **Figure S2. Optimal temperature for F1ab-RT-LAMP reaction**
- F1ab-rRT-LAMP reactions for detection of F1ab gene of SARS-CoV-2 were monitored by real-time measurement of turbidity (LA320c). Turbidity of >0.1 was regarded as positive because the threshold value was 0.1. Eight kinetic graphs (1-8) were yielded at different temperatures (61-68°C, 1^o^C intervals) with target template at the level of 1.4×10^3^ copies (F1ab-plamid) per reaction. The graphs from 63°C to 66°C showed faster amplification.
-
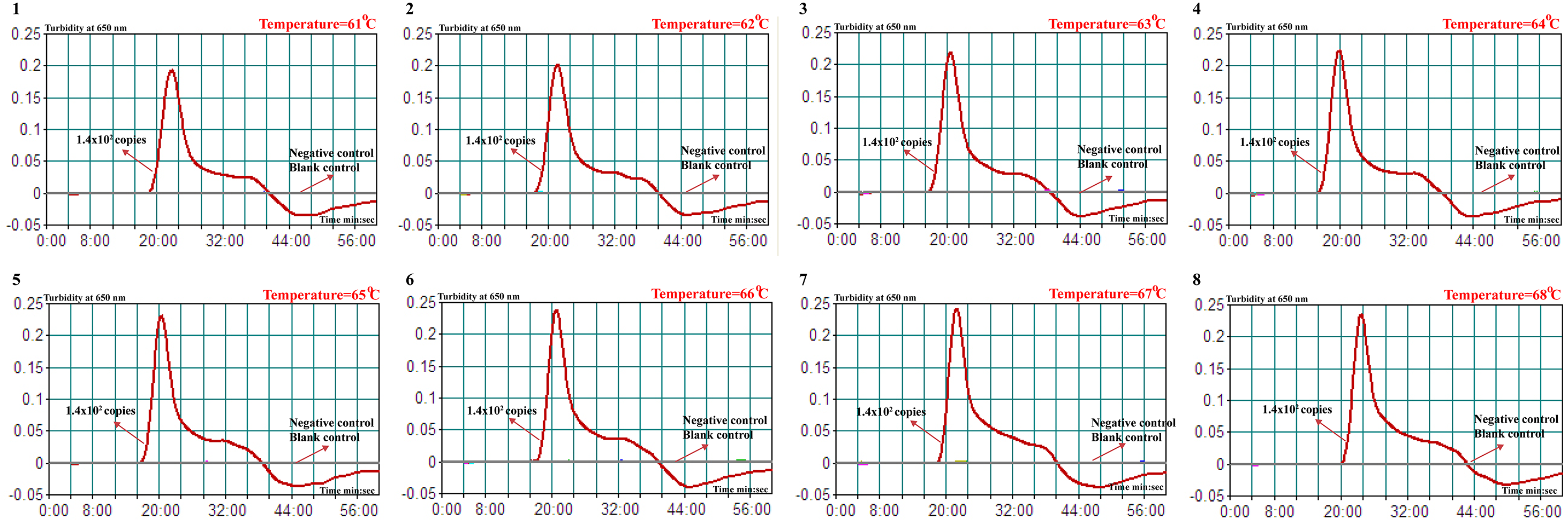

- **Figure S3. Optimal temperature for NP-RT-LAMP reaction**

The NP-rRT-LAMP reactions for detection of NP gene of SARS-CoV-2 were monitored by real-time measurement of turbidity (LA320c). Turbidity of >0.1 was regarded as positive because the threshold value was 0.1. Eight kinetic graphs (1-8) were yielded at different temperatures (61-68°C, 1^o^C intervals) with target template at the level of 1.4×10^3^ copies (NP-plamid) per reaction. The graphs from 63°C to 66°C showed faster amplification.
